# Nerve Regeneration Effect of a Composite Bioactive Carboxymethyl Chitosan-Based Nerve Conduit with a Radial Texture

**DOI:** 10.3390/molecules27249039

**Published:** 2022-12-18

**Authors:** Yijie Zhang, Zhiwen Jiang, Yanting Wang, Lixin Xia, Shuqin Yu, Hongjian Li, Wei Zhang, Wanshun Liu, Kai Shao, Baoqin Han

**Affiliations:** 1 Laboratory of Biochemistry and Biomedical Materials, College of Marine Life Sciences, Ocean University of China, Qingdao 266003, China; 15020050785@163.com (Y.Z.); jiangzhiwen@ouc.edu.cn (Z.J.); 17860751735@163.com (Y.W.); xialixinzzz@163.com (L.X.); yu.shuqin@outlook.com (S.Y.); 21180631156@stu.ouc.edu.cn (H.L.); zhangwei0515@outlook.com (W.Z.); wanshunliu@hotmail.com (W.L.); 2 Laboratory for Marine Drugs and Bioproducts, Pilot National Laboratory for Marine Science and Technology, Qingdao 266235, China; 3 Department of Central Laboratory, Qilu Hospital (Qingdao), Cheeloo College of Medicine, Shandong University, Qingdao 266035, China

**Keywords:** chitosan, carboxymethyl chitosan, nerve conduit, biocompatibility

## Abstract

Chitosan (CTS) has been used as a nerve guidance conduit (NGC) material for bridging peripheral nerve defects due to its biocompatible, biodegradable, and non-toxic properties. However, the nerve regeneration effect of chitosan alone is restricted due to its inadequate biological activity. Herein, a composite, bioactive chitosan based nerve conduit, consisting of outer warp-knitted tube scaffold made from medical-grade chitosan fiber, and inner porous cross linked carboxymethyl chitosan (C-CM-CTS) sponge with radial texture was developed. The inner wall of the scaffold was coated with C-CM-CTS solution. CM-CTS provided favorable bioactivities in the composite chitosan-based nerve conduit. An in vitro study of CM-CTS revealed its satisfying biocompatibility with fibroblast and its inhibition of oxidative damage to Schwann cells. As the internal filler of the NGC, the lyophilized sponge of C-CM-CTS showed a longitudinal guidance effect for nerve reconstruction. After 10 mm defect in rat sciatic nerve was bridged with the composite bioactive chitosan-based nerve conduit, the nerve conduit was able to effectively promote axonal regeneration and played a positive role in inducing nerve regeneration and functional recovery. In addition to the functional advantages, which are equal to those of an autograft; the technology for the preparation of this conduit can be put into mass production.

## 1. Introduction

Peripheral nerve injury repair has always been a challenge in clinical technology. Peripheral nerves have a certain degree of spontaneous regeneration ability, unlike central nerves. In 1873, Hueter directly butted and sutured the proximal and distal ends of the transverse nerve, creating the epineurium suture. End-to-end suturing cannot be used to repair a nerve defect when it involves damage over a long distance, because of the secondary damage caused by the end-to-end suturing [1,2,3]. Autologous nerve graft repair remains the gold standard [4,5]. However, it requires more surgery, and the supply of donated nerves is limited. Therefore, peripheral nerve repair is a unique challenge and opportunity for clinical and translational neuroscience. In the last few decades, various artificial nerve guidance conduits (NGCs) have been compared with autografts in the field of nerve regeneration and functional recovery [6,7,8]. The biodegradability, biocompatibility, and economic availability of natural polymers make them particularly attractive for applications in neuro-regenerative medicine [9].

Chitosan (CTS) is a kind of amino polysaccharide that is extracted from shrimp and crab shells, and its molecular composition is similar to that of human glycosaminoglycan. Chitosan has been widely used in biomedical materials because of its non-toxic, biocompatible, and biodegradable properties. In recent years, progress has been made in the application of chitosan-based materials in the bridging of peripheral nerve defects [10,11,12]. In June 2014, a chitosan nerve conduit called the Reaxon Nerve Guide received FDA approval. The nerve regeneration effect of chitosan alone was limited due to its longer degradation rate and lower tenacity [13]. Furthermore, hollow nerve ducts can cause the scattered growth of axons in the process of nerve regeneration, and indirectly lead to incorrect pairing and multi-nerve innervation in the process of nerve regeneration, which is not conducive to the repair of nerve function [14]. In recent years, there have been few studies on the repair of nerve injuries with a single chitosan conduit. It has been reported that an axial structure promotes cell migration and guides neurite growth along the axial directions [15].

Derivatives of chitosan with different structures and properties can be obtained through chemical modifications. Carboxymethyl chitosan (CM-CTS) one of the most important water-soluble derivatives of chitosan, and it has favorable biological activities in cells and in the repair of damaged tissue. CM-CTS displayed a positive influence on the stimulation of cell proliferation and neo-vascularization [16]. More importantly, vascularization provides nutrients for nerve regeneration. In addition, CM-CTS can interact with cells, which successfully results in cell growth/tissue regeneration and wound healing [16]. He et al. demonstrated the anti-apoptotic effects of CM-CTS on Schwann cells [17]. Wang et al. produced CM-CTS hollow nerve tubes with a mold casting/lyophilizing method, and a successful nerve regeneration process was achieved [18]. In previous studies, the CM-CTS fibers served as internal fillers of chitin conduit to fabricate composite nerve graft, and proved potent in repairing peripheral nerve injury [13]. CM-CTS has excellent potential for nerve repair. However, the duration time of CM-CTS is not long enough to match the repair cycle. Lu et al. prepared covalently cross linked CM-CTS (C-CM-CTS) to study its degradation and effects on peripheral nerve regeneration. The results showed that the C-CM-CTS membrane enhanced the diffusion of nerf-2A cells in comparison with a chitosan membrane and provided a suitable substrate for the proliferation of nerf-2A cells [19]. Moreover, three dimensional scaffolds prepared by the freeze drying method were created to mimic native extracellular matrix [20]. In the present study, our filler lyophilized C-CM-CTS sponge has a porous and radial texture structure, which may provide more attachment sites for Schwann cells and guide nerve regeneration. Meanwhile, C-CM-CTS degrades slowly [18], and can be gradually released throughout the whole process of nerve regeneration.

Herein, a composite bioactive nerve conduit was prepared entirely based on chitosan and its derivatives. Medical-grade chitosan fibers were used as raw materials to warp knit the outer tube scaffold, which was further coated with C-CM-CTS solution. A porous C-CM-CTS sponge with a radial texture was used for the inner filling of the conduit stent in order to provide bioactive ingredients for nerve regeneration. The physical and chemical properties of the composite nerve conduit were analyzed. The biocompatibility and bioactive effects of CM-CTS were further assessed by using fibroblast L929 and Schwann cells. Finally, the composite bioactive nerve conduit was adopted for the bridging of a defected rat sciatic nerve in order to evaluate the repair effect with the aim of broadening the types of artificial nerve scaffolds. The data will provide an experimental basis for the expansion of the applications of chitosan in peripheral nerve tissue engineering.

## 2. Results and Discussion

### 2.1. Characterization of the Chitosan-Based Composite Bioactive NGC

**Figure 1:**
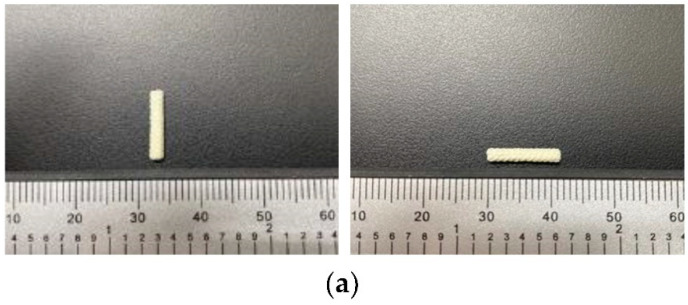

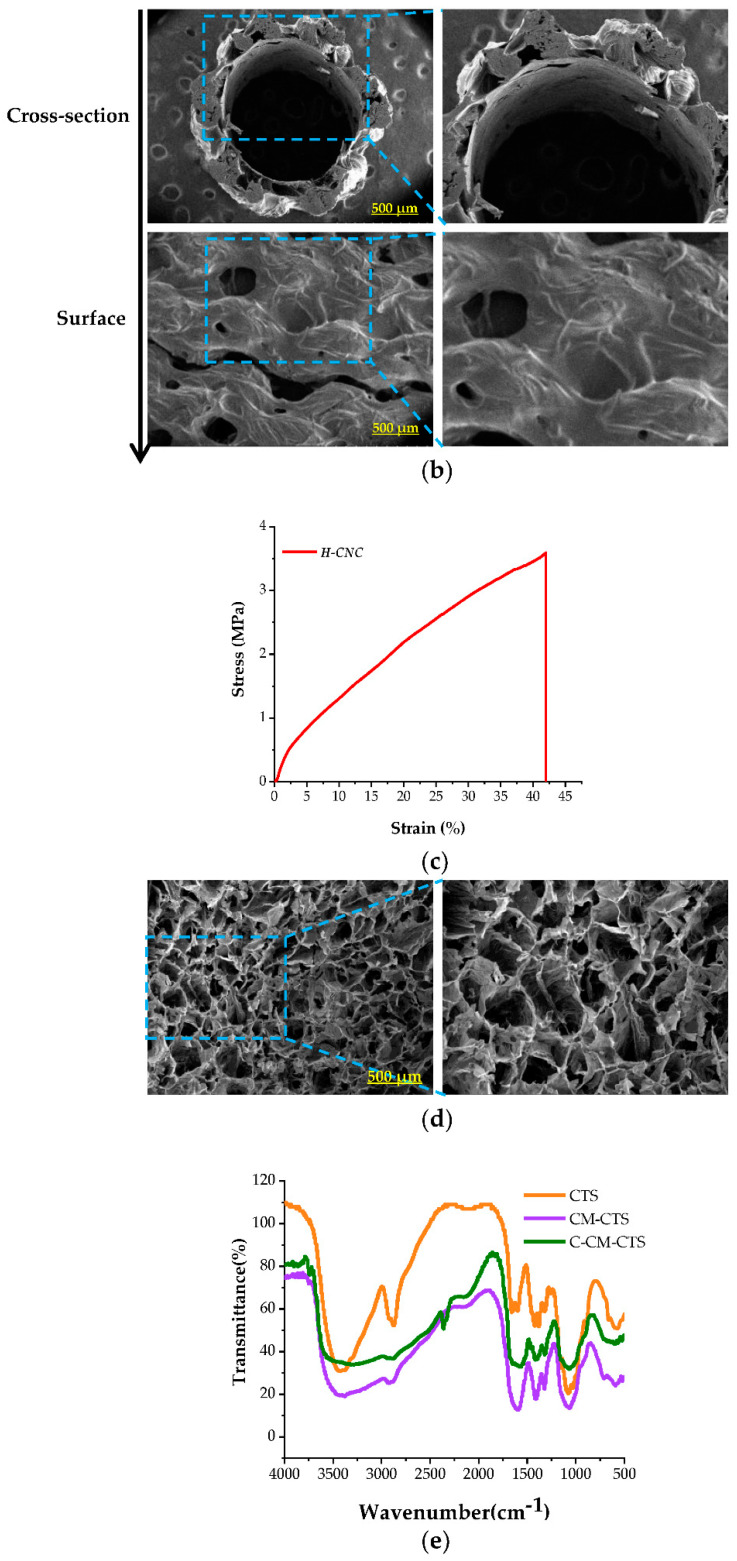
Characterization of the chitosan-based composite bioactive nerve conduit. (**a**) General view of the H-CNC (inner diameter = 2 mm). (**b**) Morphology observation of the H-CNC by SEM. (**c**) The stress-strain pattern of the H-CNC. (**d**) SEM morphology of C-CM-CTS sponges. (**e**) Fourier-transform infrared spectroscopy (FT-IR) spectra of chitosan, CM-CTS, and C-CM-CTS.

Medical chitosan fiber was used as a material for the preparation of the wall of a chitosan conduit by using the warp-knitting technique in order to increase the mechanical strength of the conduit. Freeze-dried C-CM-CTS sponges were used to fill in the conduits in order to provide better support for nerve growth. As shown in Figure 1a, the conduit that we prepared had an outer diameter of 2 mm, a wall thicknesses of 0.4 mm, and a length of 12 mm. The presence of the textile fibers and the morphologies of the surface and cross-section of the hollow chitosan-based nerve conduits (H-CNCs) were observed through scanning electron microscopy (SEM, Figure 1b). The warp-knitted structure was able to provide mechanical support and strength for the nerve conduit, and act as a barrier to prevent the growth of fibrous tissue. The porous surface enabled the exchange of nutrients and liquids. The desired NGC should not only prevent the infiltration of fibrous tissues into the conduit, but should also ensure the entry of oxygen and nutrients into the inner lumen [21]. According to the calculation of the SEM images with the Image J software, the average porosity of the nerve conduit was 69.82%, which was significantly higher than that of a traditional coated tube. The greater number of stomata facilitated the flow of tissue fluid and nutrient exchange, as well as the vascularization of regenerated nerves within the conduit [13].

The main reason for the failure of nerve repair is that the mechanical strength of the nerve conduit is insufficient to withstand the strain of the surrounding tissue after implantation [22]. The modulus of elasticity is an essential parameter in material mechanics. Sufficient mechanical properties for preventing the collapse of the nerve conduit are some of the crucial factors that must be considered in the design of artificial nerve conduits [23]. The stress–strain pattern of the chitosan based nerve conduit (Figure 1c) showed that the average elastic modulus, elongation at break, and maximum load were 3.59 MPa, 39.75%, and 10.83 N, respectively, indicating the good mechanical properties and long term stability of the tubular structure for the support of nerve regeneration. The CM-CTS composite nerve conduit (CM-CNC) was obtained from the H-CNCs that were filled with the freeze-dried CM-CTS sponge cross linked by 1, 4-budiglycidyl ether. The SEM images demonstrated that the filler C-CM-CTS sponges exhibited porous and radial texture structures, which may provide more attachment sites for Schwann cells and guide nerve regeneration (Figure 1d). The chemical structure of the C-CM-CTS was confirmed through infrared spectroscopy (Figure 1e). The absorption peaks at 1594 and 1410 cm^−1^ were the antisymmetric and symmetric stretching vibration peaks of COO-, which were the characteristic absorption peaks of CM-CTS. The absorption peak at 1064 cm^−1^, which represented the stretching vibration of the C-O that reacted with primary alcohol, confirmed that the substitution occurred mainly at the C-6 position [24]. The results of the infrared spectrum showed that the low cross linking did not affect the chemical structure of CM-CTS. Therefore, the inner wall of the scaffold was coated with C-CM-CTS solution, and a C-CM-CTS sponge with a porous and radial morphology was also adopted to fill the lumen of the chitosan composite nerve.

### 2.2. The In Vitro Biocompatibility of CM-CTS

**Figure 2:**
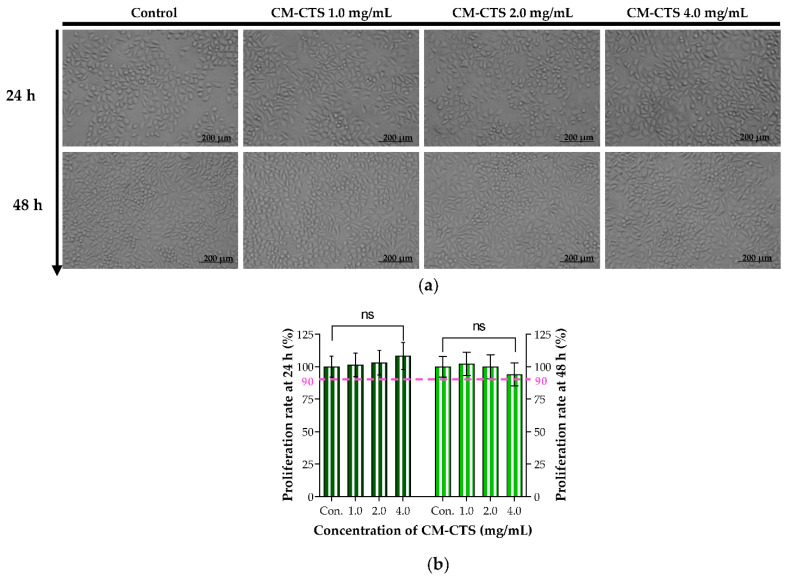
Effects of CM-CTS on the cell morphology and proliferation rate of L929 cells. (**a**) Morphological changes of L929 cells were examined under a light microscope (original magnification, 100×); (**b**) Proliferation rate of L929 cells by MTT assay at 24 and 48 h. The data are represented by the mean ± SD, *n* = 6, compared with the control group, ns: not significant.

CM-CTS is the most important degradable component of the chitosan based composite bioactive nerve conduit. With reference to “ISO 10993-5(1999)”, its effect on the growth of L929 fibroblasts was adopted in order to assess the biocompatibility of CM-CTS in vitro. After being cultured in Dulbecco’s modified Eagle’s medium (DMEM) containing different concentrations of CM-CTS for 24 and 48 h, the cell morphology of L929 in each group was assessed, as shown in Figure 2a. The L929 cells had an exemplary cellular growth behavior. They tended to have a spindle cell pattern. The cells in different groups grew well at each time point, with a uniform distribution and fusiform morphology. There was no obvious difference in cellular growth status between the CM-CTS-treated groups and the control group. Our findings showed that the cells in contact with the CM-CTS displayed similar cell growth states with the normal cells, and they kept the typical cellular morphological characteristics and spreading on a large scale. As shown by the MTT data in Figure 2b, the proliferation rates of L929 cells in the CM-CTS treatment groups (the concentration of 1.0–4.0 mg/mL) were all over 100% at 24 h and were greater than 90% at 48 h, which met the ISO 10993-5 Standard and thus indicated biocompatibility and non cytotoxicity of CM-CTS in vitro. Furthermore, the proliferation rates of L929 cells is dose dependent after treatment with CM-CTS for 24 h, while the cell proliferation rate was the lowest (94.11%, 48 h) in the CM-CTS group at 4.0 mg/mL because of the high viscosity of the solution, which was consistent with Chang’s study [25].

Chitosan-based conduits can reduce extensive scarring [26,27] and prevent the formation of neuromas after peripheral nerve injury [28]. In the present study, chitosan and C-CM-CTS were chosen for the fabrication of nerve conduits with a warp-knitting technology to provide the conduits with mechanical properties, processability, and biocompatibility that could meet the demands of the reconstruction of damaged nerves. The regeneration potential of peripheral nerves is attributed to the flexible differentiation properties of Schwann cells, which can transform into repair cells after injury [29]. After assessing its biocompatibility with L929 cells, the bioactive effects of CM-CTS were further evaluated with Schwann cells.

### 2.3. Protective Effect of CM-CTS on Hydrogen-Peroxide-Damaged Schwann RSC96 Cells

Schwann cells play an essential role in maintaining the normal physiological functions of axons, and in the process of nerve regeneration [30]. Damage to Schwann cells is likely to induce cell apoptosis and restrict the functional recovery of peripheral nerves [31]. As reported in previous studies, apoptosis of Schwann cells caused by oxidative stress is a common and vital mechanism of peripheral neuropathy [32]. Hydrogen peroxide (H_2_O_2_) is a reactive oxygen species that causes cell toxicity and induces apoptosis in multiple types of cells [33]. Thus, inhibiting oxidative damage to Schwann cells improves the potential protective and regenerative effects in the case of peripheral nerve injury. Herein, a H_2_O_2_-induced cell damage model was used to investigate the potential cytoprotective activity of CM-CTS on RSC96 cells through a morphological study, MTT assay, and Hoechst 33258 staining.

**Figure 3:**
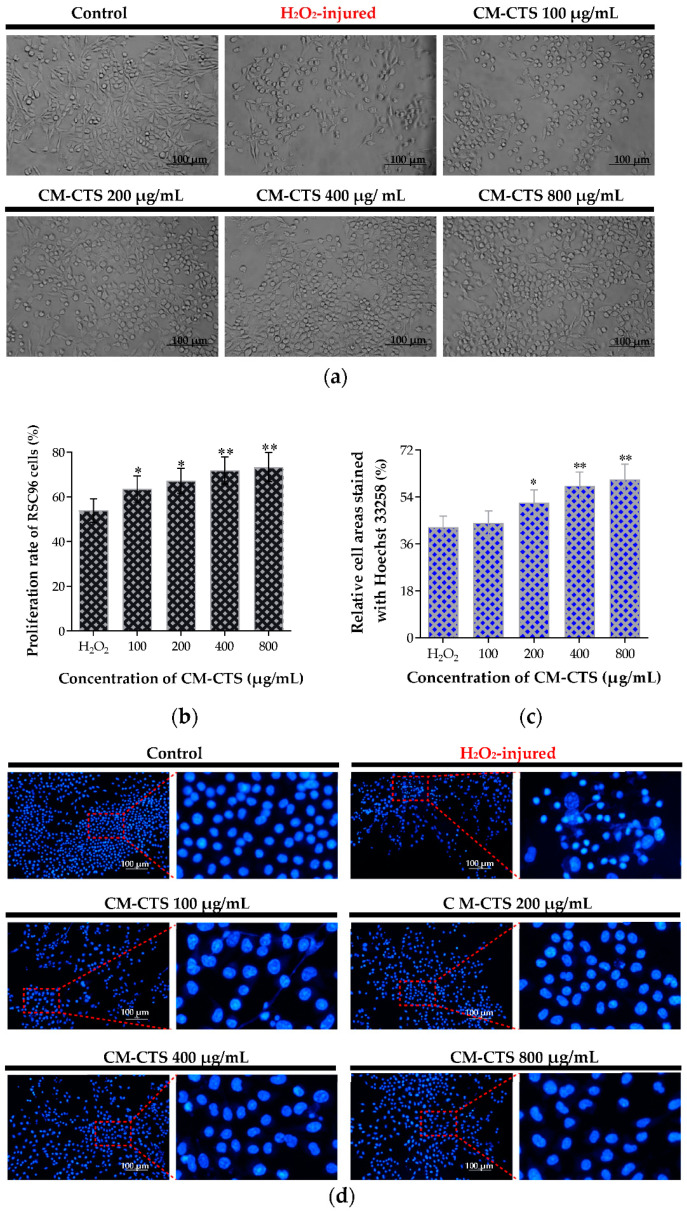
Protective effect of CM-CTS on RSC96 cells damaged by hydrogen peroxide. (**a**) Cell morphology of RSC96 (original magnification, 100×); (**b**) Proliferation rate of RSC96 cells measured with MTT assay; (**c**) Relative cell areas stained with Hoechst 33258 (%); (**d**) Hoechst 33258 (blue) was used to stain the nuclei. The data are represented by the mean ± SD, *n* = 6, * *p* < 0.05, ** *p* < 0.01 significant difference in comparison with the H_2_O_2_-injured group.

Observations of the cell morphologies in the control group, H_2_O_2_-injured group, and CM-CTS treatment groups were displayed in Figure 3a. The RSC96 cells began to contract after the addition of H_2_O_2_, resulting in decreased cell areas and increased intercellular spaces. Interestingly, there were distinct differences in the cell growth status between the CM-CTS-pretreated cells and H_2_O_2_-injured cells. The CM-CTS pretreated cells presented a higher cell density compared to the H_2_O_2_-injured cells, and it was a dose-dependent process. In addition, as exhibited in Figure 3b, the proliferation rates of CM-CTS-pretreated RSC96 cells were significantly higher than those of H_2_O_2_-injured cells, and the apparent cytoprotective effect was observed in a dose dependent manner. To further examine the protective activity of CM-CTS in RSC96 cells against H_2_O_2_-induced apoptosis, fluorescent nuclear staining was performed, and the microscopic observation and quantified results were shown in Figure 3c,d. After the Hoechst 33258 staining, the nuclei of normal cells were blue. Still, the apoptotic nuclei were atrophied and fragmented. In comparison with the H_2_O_2_-damaged group alone, pretreatment with CM-CTS significantly increased the number of healthy cells, and this concentration at 800 μg/mL worked the best according to the quantified cell areas data. These data suggested that CM-CTS prevented the Schwann RSC96 cells from undergoing oxidative-stress-induced damage. He’s study also showed that CM-CTS markedly rescue the apoptotic morphology in H_2_O_2_ treated primary Schwann cells drawn from rat sciatic nerve in dose-dependent manner (50–200 μg/mL) [34]. Thus, the lyophilized sponge of C-CM-CTS was used as the filling material for the lumen, which allowed the bioactive requirements of the nerve conduits to be fully met.

### 2.4. Functional Recovery of the Sciatic Nerve after a CM-CNC Transplant

**Figure 4:**
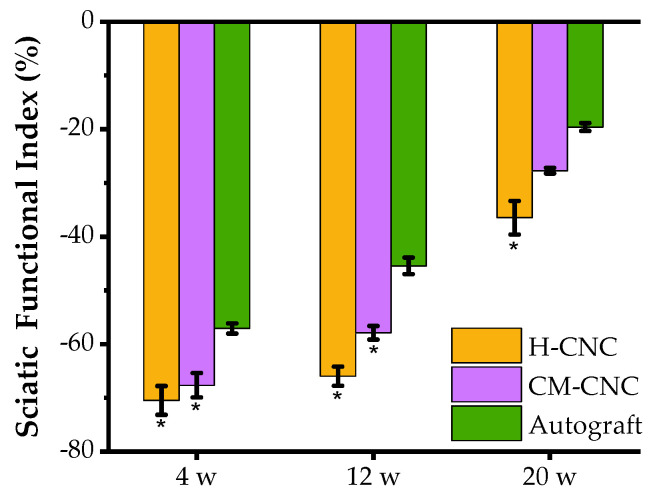
Rat sciatic nerve function index in each group. The data are represented by the mean ± SD, *n* = 6, * *p* < 0.05 significant difference in comparison with the autograft group.

Indexing of the sciatic function through gait analysis is essential for the assessment of the recovery of lower limb motor function in rats. The value of the sciatic functional index (SFI) ranges from –100 to 0, with 0 representing normal function and –100 representing complete injury [35]. With the recovery of nerve function and muscle function, the SFI progressively improved and exercise ability was gradually recovered. In comparison with the data at 12 w, the SFI was remarkably increased in all groups at 20 w, except in the H-CNC group (Figure 4). The SFI in the CM-CTS composite nerve conduit (CM-CNC) group had no significant differences from that in the autograft group (*p* > 0.05). Obviously, the sciatic nerve function index of the CM-CNC group was significantly better than that of the H-CNC group. Subsequently, the CM-NGC showed significant improvements in the SFI at 20 w, with a value equal to that of the autograft.

### 2.5. Electrophysiological Recovery of a Damaged Nerve after a CM-CNC Transplant

**Figure 5:**
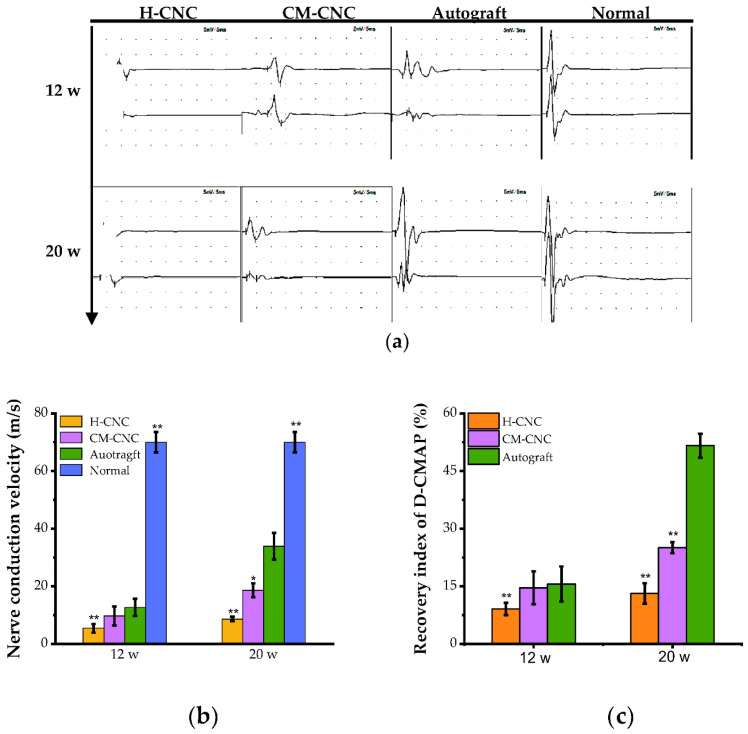
Electrophysiological evaluation of the regenerated sciatic nerve. (**a**) CMAP of regenerated nerve. (**b**) Regenerative nerve conduction velocity (m/s). (**c**) Maximum amplitude recovery index of CMAP (%). The data are represented by the mean ± SD, *n* = 6, * *p* < 0.05, ** *p* < 0.01 significant difference in comparison with the autograft group.

Nerve conduction velocity (NCV) and compound muscle action potential (CMAP) are essential criteria for evaluating nerve regeneration. The amplitude of the CMAP, an indicator of nerve conduction, is directly proportional to the number of nerve fibers that can effectively conduct nerve impulses in the measured sciatic nerve trunk [36,37]. At 12 and 20 w, CMAP recordings were used to display the electrophysiological performance of rats (Figure 5a). As expected, the motor NCV and CMAP amplitude of the injured sides were dramatically weaker than those of the contralateral normal sides. However, the NCV and CMAP amplitude of the autograft group were considerably higher than those of the H-CNC and CM-CNC groups at 12 w (Figure 5b,c). In particular, with the growth of the regenerated nerves in the H-CNC and CM-CNC groups, the electrical signal was gradually recovered, showing an increase in CMAP and NCV at 20 w. From 12 to 20 w, the operative latency of the rats in each group became shorter, and the NCV and maximum amplitude showed an increasing trend. The above data indicated that the regenerated nerve was enhanced, and the chitosan based composite nerve conduit was able to promote the repair of defects in the sciatic nerve. The electrophysiological results of the CM-CNC group were superior to those of the H-CNC group, indicating that the chitosan based composite nerve conduit filled with the C-CM-CTS sponge had a better effect on the promotion of nerve repair.

### 2.6. Morphological and Ultrastructural Changes in Repaired Nerves by CM-CNC

**Figure 6:**
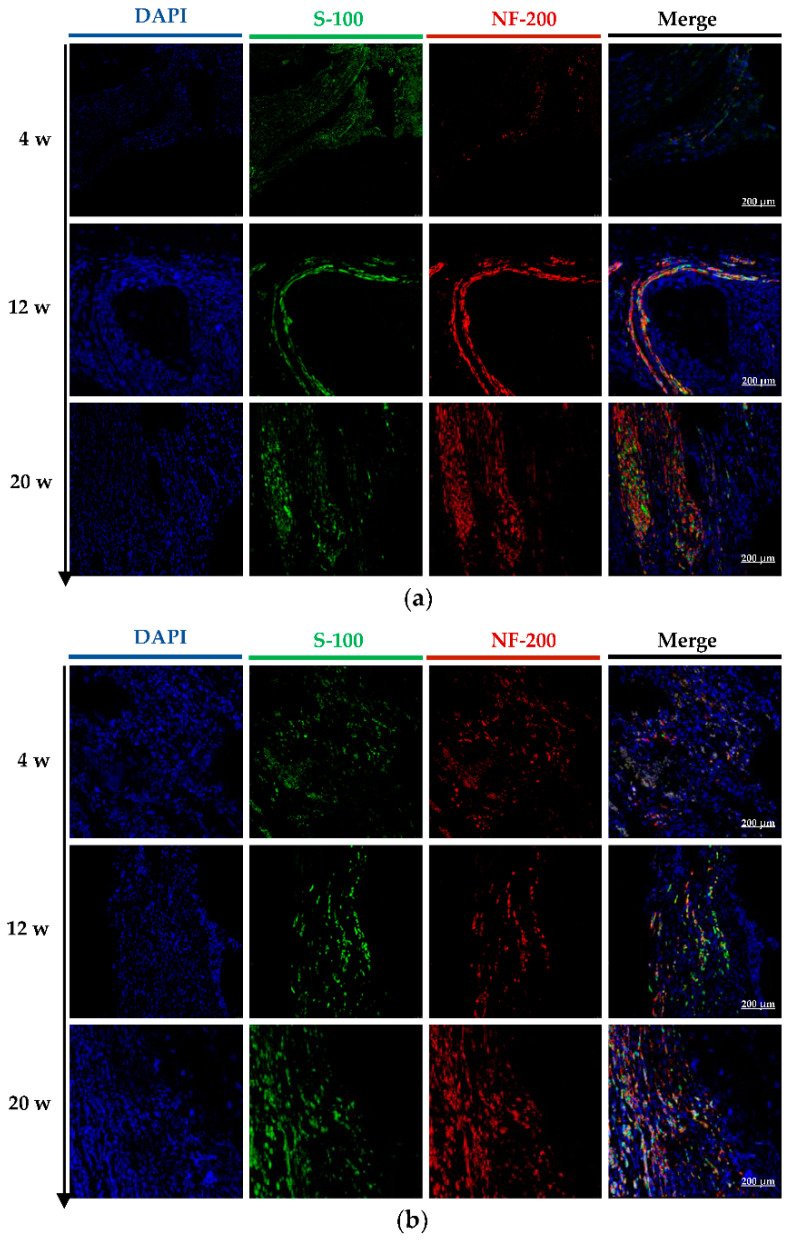

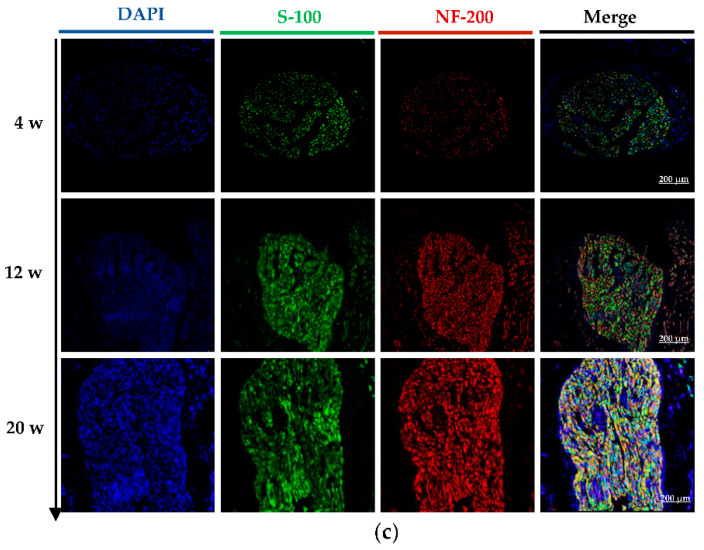
The observation of regenerated nerves immunofluorescence staining by laser confocal microscope in the H-CNC group (**a**), CM-CNC group (**b**), and autograft group (**c**).

Double labeled NF-200 and S-100 were observed through laser confocal microscopy on the longitudinal sections of regenerated nerves at 4, 12, and 20 weeks post operation (Figure 6a–c). At 4 w, positive green labels of S-100 and a small number of positive red labels of NF-200 were observed in the H-CNC group and CM-CNC group. The positive signals indicated the presence of regenerated nerves, but the regenerated nerve fibers were incomplete. Similarly, positive green labels of S-100 and a small number of positive red labels of NF-200 were observed in the autologous group, showing some atrophy of the autograft nerve. At 12 w, the number of positive green labels of S-100 and positive red labels of NF-200 in the longitudinal section of the renewable nerve trunk observably increased in the H-CNC and CM-CNC groups, indicating that the regeneration of Schwann cells and the nerve trunk was vigorous; in particular, the regenerated nerve fibers in the CM-CNC group were bunched and evenly distributed. The positive density of S-100 and NF-200 in the autograft group was dramatically elevated, indicating that the autograft nerve was repaired well. At 20 w, the density of Schwann cells in the H-CNC group and the CM-CNC group was further increased, and there was a large number of nerve fiber bundles; the nerve fibers in the CM-CNC group were denser than those in the H-CNC group. A histological observation provided an exciting comparison, which showed that the density and length of the regenerated nerve fibers in the CM-CNC and autograft groups were significantly higher than those in the H-CNC group, and they were similar between the CM-CNC group and autograft group.

Schwann cells, an excellent growth-promoting substrate providing regenerated axons with many neurotrophic factors, cell adhesion molecules, and other factors involved in axon growth and guidance, play an essential role in the tissue structure of peripheral nerves [23]. Because the hollow nerve conduit did not improve the adhesion site of Schwann cells in the process of nerve regeneration, it was found that the number of Schwann cells in the central position of the hollow nerve conduit was lower than that in the filler group. Our results also suggested that the use of the porous C-CM-CTS sponges as inner fillers of composite nerve conduits could effectively guide and promote nerve reconstruction.

**Figure 7:**
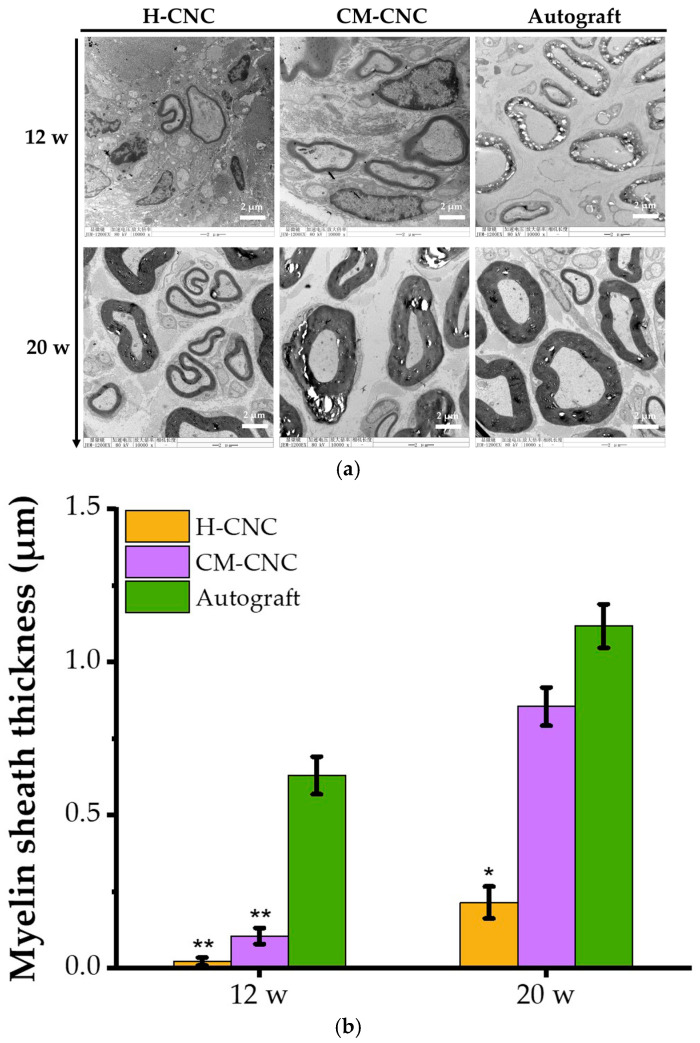
Effects of the CM-CNC on ultrastructural changes in regenerated nerves. (**a**) Observation of the ultrastructure of regenerated nerves through transmission electron microscopy (10,000×). (**b**) The thickness of the regenerated myelin sheath (μm). The data are represented by the mean ± SD, *n* = 6, * *p* < 0.05, ** *p* < 0.01 significant difference in comparison with the autograft group.

The ultrastructure of the regenerated nerves was observed through transmission electron microscopy (TEM) at 12 and 20 w (Figure 7a). At 12 w, the myelin sheaths in the autologous group were larger and more regular, and the myelin sheaths were thicker than those in the H-CNC and CM-CNC groups. By contrast, early embryonic nerve fibers appeared in the CM-CNC group with a small diameter and irregular shape, thin myelin wall, sparse but many regenerated myelinated nerve fibers, and a small number of nerve fiber embryonic fibers also appeared in the H-CNC group. At 20 w, the autogenous group had good growth, and the myelin wall was significantly thickened and more regular. The myelin walls in the H-CNC group and the CM-CNC group were more regular than those at 12 w, with obvious axons, a larger diameter of myelinated nerve fibers, and a significantly thickened myelin wall, and the results were more significant in the CM-CNC group. The wall thickness of myelinated nerve fibers is crucial for the estimation of the maturity degree of regenerated nerve fibers [38]. The myelin wall thickness of each group was statistically analyzed with the Image J software (Figure 7b). The analysis further indicated the myelin wall thickness of the H-CNC group and CM-CNC group was distinctly less than that of the autologous group at 12 w (*p* < 0.01). The myelin wall thickness of the CM-CNC group at 20 w was markedly greater than that of the H-CNC group, and there were no significant differences between the CM-CNC group and the autologous group (*p* > 0.05), which further confirmed the effect of the chitosan-based composite nerve conduit on the promotion of nerve regeneration.

### 2.7. Muscle Wet Weight and Motor Endplate Analysis after the CM-CNC Transplant

**Figure 8:**
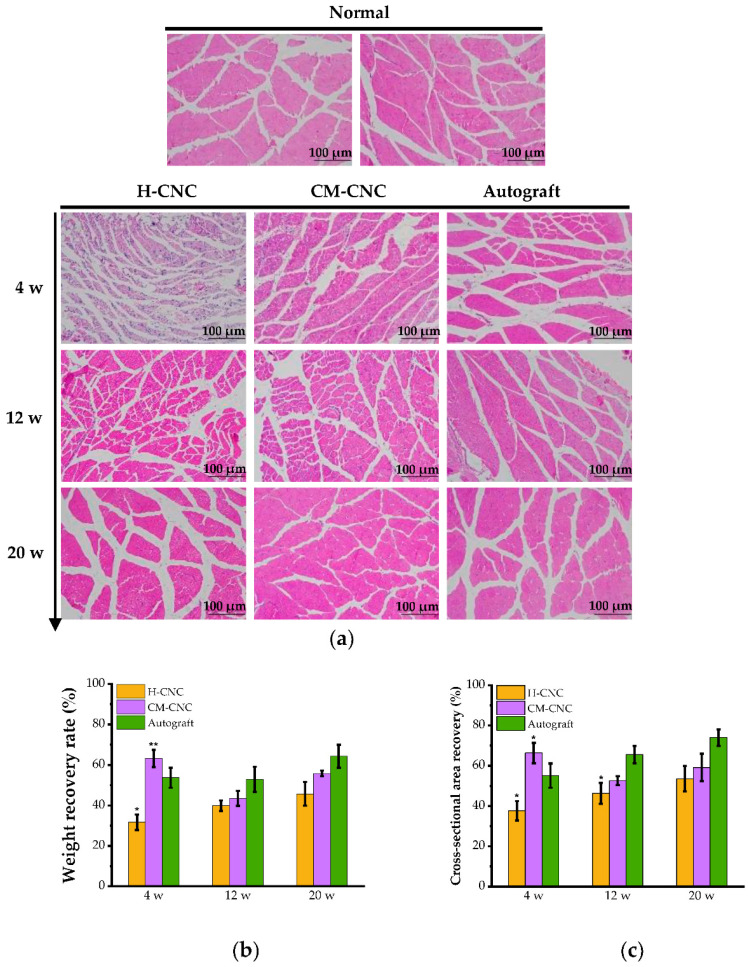
HE-stained images and wet weight of the gastrocnemius. (**a**) Morphology of the gastrocnemius muscle according to HE staining in each group. (**b**) The wet-weight ratio of gastrocnemius muscles. (**c**) Cross-sectional area recovery index of muscle (%). Data represent mean ± SD, *n* = 6, * *p* < 0.05, ** *p* < 0.01 significant difference compared with the autograft group.

The gastrocnemius muscle index (GMI) value and pathological structure of the gastrocnemius muscles on the injured side and uninjured sides were obtained with the bilateral weight ratio and hematoxylin–eosin (HE) staining to evaluate the degree of muscular atrophy. The wet weight of the gastrocnemius muscle decreased significantly at 4 w due to muscular atrophy, and the atrophy in the H-CNC group was the most severe. On the contrary, it recovered gradually at 12 and 20 w, and the recovery rates of the H-CNC, CM-CNC, and autogenous groups were 46.8%, 55.8%, and 62.7%, respectively (Figure 8b). The HE staining results at 4 w after surgery showed muscle fiber atrophy and disordered arrangement in both the H-CNC group and CM-CNC group, and the recovery rates of the muscle cross-sectional area were remarkably different than that in the autogenous group (*p* < 0.05). Muscle fiber atrophy was still present at 12 w, but less so in the CM-CNC group than in the H-CNC group, and it recovered gradually at 20 w. The shape and size of muscle fibers in the autogenous group were similar to those on the normal side. The shape, size, and recovery of muscle fibers were significantly increased in the H-CNC and CM-CNC groups, and they were more significant in the CM-CNC group (Figure 8a). The wet weight ratio and muscle cross-sectional area recovery rate were basically the same, and they were significantly higher in the CM-CNC group than in the H-CNC group (Figure 8c).

The functional recovery of the regenerated nerves was assessed via behavioral tests, as well as through electrophysiological and morphological analyses. Regeneration of the myelinated nerve fibers was shown in the distal end of the injured nerves of rats treated with chitosan conduits with in-wall micro patterning [12]. More myelinated nerve fibers were formed in the injured rat nerves that were treated with the CM-CNC than in the injured rat nerves that were treated with the H-CNC. As is commonly known, the degree of muscular atrophy is an important indicator of nerve function regeneration, and it can be used to further evaluate neural regeneration together with the histological structure [39]. In this study, the beginning of the innervation of the target muscle was noticed, indicating that the target muscle acquired the ability to recover its nerve function. These results correspond with the electrophysiological differences in the regenerated nerves among the three grafted groups. In addition, the functional tests and morphometric analyses also provided quantitative data that demonstrated that no significant differences in nerve regeneration were found between rats treated with a nerve conduit or autologous nerve graft.

## 3. Materials and Methods

### 3.1. Materials and Reagents

Medical chitosan fibers and CM-CTS (degree of carboxylation 87.6%) were obtained from Qingdao Biotemed Biomaterial Co., Ltd. (Qingdao, China). 1,4-Butanediol diglycidyl ether was purchased from Aladdin Biochemical Technology Co., Ltd. (Shanghai, China). MTT was purchased from Sigma-Aldrich (St. Louis, MO, USA). Anti-S-100 antibody and anti-NF-200 antibody were bought from Abcam Inc (Cambridge, MA, USA). DAPI, Hoechst 33258, and trypsin were offered by Gibco^®^, Life Technologies (Carlsbad, CA, USA).

### 3.2. Cell Lines and Animals

The mouse fibroblast (L929) and rat Schwann cells (RSC96) were obtained from the Cell Bank of Chinese Academy of Science (Shanghai, China). The complete culture medium was high glucose, DMEM enriched with fetal bovine serum (10%, *v*/*v*), streptomycin (100 μg/mL) and penicillin (100 U/mL). With the animal license number SCXK (Yu) 2019-0002, Sprague-Dawley (SD) rats (220 ± 20 g) were obtained from Huaxing Laboratory Animal Co. (Zhengzhou, China). All experimental protocols followed the National Research Council’s Guide for the Care and Use of Laboratory Animals. Ethical approval to conduct the protocol was granted by the ethics committee at Ocean University of China (approval number: OUC-AE-2021-157)

### 3.3. Preparation of the Chitosan-Based Composite Bioactive Nerve Conduit

Chitosan nerve conduit scaffolds with an inner diameter of 2 mm were made from medical chitosan fiber by using a spinning machine. The conduit scaffolds were immersed in 0.2 mol/L NaOH solution to remove impurities, washed with distilled water to remove lye, and dehydrated with alcohol. The inner and outer surfaces of the conduit scaffolds were coated with CM-CTS solution containing 1,4-butanediol diglycidyl ether (BDDE) that was cross linked for 12 h and dried to obtain hollow chitosan based nerve conduits (H-CNCs). The CM-CTS solution containing BDDE was freeze dried into fibrous porous sponge materials, which were used to fill the chitosan based nerve conduits to obtain chitosan based composite nerve conduits (CM-CNCs).

The Fourier-transform infrared spectroscopy (FT-IR) spectra of cross linked CM-CTS (C-CM-CTS), CM-CTS, and chitosan were analyzed with a Nicolet AVATA 370. The sample was ground, dried in a vacuum, mixed with KBr powder, and compressed into discs for the FT-IR examination. The cross section and surface morphology of the H-CNCs were assessed through SEM. The samples were coated with gold by using a JEOL JFC-110E Ion Sputter before examination with SEM. The mechanical properties of the H-CNCs were assessed on an AGS-X universal testing machine (Shimadzu, Suzhou, China) [13], and the H-CNCs were fixed to clamps at both ends of the tension machine. A stretching speed of 10 mm/min with the set effective length of 10 mm for the H-CNCs was used until the sample was fractured. The maximum load N and the tensile length L were recorded simultaneously to calculate Young’s modulus.

### 3.4. Biocompatibility of CM-CTS In Vitro

Mouse fibroblast L929 cells were cultured in contact with the CM-CTS solution to evaluate its biocompatibility through MTT assay. L929 cells were inoculated into 96-well plates at an initial density of 1.5 × 10^4^/well in a 200 μL culture medium overnight. Subsequently, the original medium was replaced with various levels (1.0, 2.0, and 4.0 mg/mL) of CM-CTS solutions. After 24 and 48 h, the cell morphology was recorded with a T1-SM 100 inverted microscope (Nikon Corporation, Tokyo, Japan). The relative proliferation rate of fibroblasts was examined with the MTT method, as previously described [40,41].

### 3.5. Bioactive Effects of CM-CTS In Vitro

Hydrogen peroxide (H_2_O_2_) was used to establish an oxidative damage model in Schwann RSC96 cells. RSC96 cells were inoculated into 96-well plates at a density of 1.5 × 10^4^/well in a 200 μL culture medium overnight. Subsequently, the original medium was replaced with various levels (100, 200, 400, and 800 μg/mL) of CM-CTS solutions for 24 h. Then, the pre-treated cells were damaged with H_2_O_2_ for 24 h. Finally, the cell morphology was recorded with a T1-SM 100 inverted microscope (Nikon Corporation, Tokyo, Japan). The relative proliferation rate of the Schwann cells was examined with an MTT assay, and Hoechst 33258 fluorescence staining was performed to observe the cell viability of the damaged RSC96 cells.

### 3.6. Ten-Millimeter Sciatic Nerve Defect in SD Rats

Adult female SD rats were randomly divided into three groups: the H-CNC group, CM-CNC group, and autologous nerve group. The rats were anesthetized by injecting sodium pentobarbital solution (30 mg/kg body weight). After shaving and disinfection, the sciatic nerve was exposed in the left lateral thigh, and an 8 mm segment was excised and slightly retracted to form a 10 mm gap at the distal and proximal stumps. In the H-CNC group and the CM-CNC group, the nerve space was bridged with an H-CNC and CM-CNC, respectively, between the proximal and distal ends of the transverse sciatic nerve space, and both ends of the catheter (1 mm at each end) were surgically sutured. In the autograft group, the transected nerve segment was surgically linked to the nerve space again. Following nerve transplantation, the muscle layer and skin were sutured, and the surgical incision was closed for routine procedures. All rats were fed separately and intraperitoneally injected with penicillin once per day for one week after the operation.

### 3.7. Behavioral Analysis

The use of gait analysis technology to investigate the motor function of the lower limbs of rats is an important method for the evaluation of the recovery of peripheral nerve injury, with the sciatic function index being the main index for observation. SFI was calculated for each group to determine the motor recovery following implantation at 4, 12, and 20 w before sacrifice. Briefly, the rats were put into a self-made footprint walking box to record the shape of the footprint, which was a wooden trough with a length of 70 cm, a width of 10 cm, and a height of 10 cm. The parameters obtained from the rats’ footprints were the middle toe spread (IT), toe spread (TS), and print length (PL) of both the experimental (E) and normal (N) hindquarters. SFI was calculated with the following formula [42]: SFI = 13.3 × (EIT-NIT)/NIT + 0.95 × (ETS-NTS)/NTS − 38.3 × (EPL-NPL)/NPL − 8.8.

### 3.8. Electrophysiological Measurements

Electrophysiological assessments of the rats were performed at 12 and 20 w. Before the electrophysiological examination, under anesthesia with a compound anesthetic, the sciatic nerve at the original operation site was re-exposed. Electrical stimulation (intensity of 10 mA, pulse width of 0.01 ms) stimulated the proximal and distal ends of the graft in turn. The CMAPs were automatically recorded in the abdomen of the gastrocnemius. A similar assessment was performed on the uninjured contralateral side. Based on the spreads between the proximal and distal stimulated parts, the motor NCV was calculated [23].

### 3.9. Immunofluorescence Analysis

By the end of the study in each time point (4, 12, and 20 w), the SD rats were sacrificed under deep anesthesia. The immunofluorescence technique was used on the midsection of the graft. After deparaffinization of the paraffin sections, endogenous peroxidase activity was quenched by incubation with 3% hydrogen peroxide for 10 min. Heat-induced antigen retrieval in the solution of sodium citrate (pH 6.0) was then performed with a microwave oven (100 °C, 10 min), and unspecific binding was blocked with BSA buffer (5% in phosphate-buffered saline). Primary mouse anti-NF-200 (1:400 dilution) and rabbit anti-S-100 (1:400 dilution) were incubated at 4 °C for one night, and then fluorescence labeled secondary antibody was reacted for one hour at 37 °C and avoided light. Finally, nuclei were counterstained with DAPI. All slides were sealed with the addition of an anti-fluorescence quenching agent and observed by using a laser scanning confocal microscope.

### 3.10. TEM Observation

The nerve regeneration in the middle of the graft was investigated through the TEM. The sample was set in pre-frozen 2.5% glutaraldehyde for 3 h, fixed with 1% osmium tetroxide, and embedded in Epon812. Myelin regeneration was observed under a TEM (HT7700, Hitachi, Ltd., Tokyo, Japan). Meanwhile, ImageJ software was used to assess the myelin sheath thickness.

### 3.11. Analysis of Target Muscles

GMI was used to assess the degree of muscle-mass recovery [43]. GMI was evaluated by using the muscle wet-weight ratio and the cross-sectional area of the muscle. At 4, 12, and 20 weeks post operation, the gastrocnemius muscles of both the experimental and normal sides were completely isolated for muscle wet-weight measurement. The middle part of each gastrocnemius was further fixed with 4% paraformaldehyde, embedded in paraffin, and stained with HE. The HE-stained gastrocnemius slices were subjected to morphometric analysis. Statistical analysis was performed on the normal side and the operative side of the rats every week. Four sections were selected for each sample, and three high-power fields were randomly chosen for each section. The images obtained were used to measure the muscle cross-sectional area of each sample with Image J.

### 3.12. Statistical Analysis

All statistics were performed by independent sample t-test or one-way analysis of variance (ANOVA). * *p* < 0.05 is considered statistically significant, and ** *p* < 0.01 is considered highly significant.

## 4. Conclusions

A CM-CTS-based composite bioactive nerve guidance conduit for peripheral nerve regeneration was successfully prepared via the warp knitting technique, and the compound nerve conduit was obtained by using a cross linked CM-CTS sponge to fill a chitosan based nerve conduit. CM-CTS was non cytotoxic to L929 cells and showed increased biocompatibility in vitro. In addition, CM-CTS markedly protected Schwann cells against H_2_O_2_-induced oxidative damage and apoptosis, which was beneficial for the improvement of the potential protective and regenerative effects on injured peripheral nerves. Furthermore, the CM-CTS-based nerve conduit was used to bridge a 10-mm-long sciatic nerve defect in rats. The functional and histological assessments provided evidence that the CM-CTS-based nerve conduit was able to significantly promote the nerve regeneration and nerve function recovery in peripheral nerve defects, which may hold a promise for future therapies for peripheral nerve injury.

## Data Availability

Not applicable.

## Sample Availability

Samples of the compounds are not available from the authors.

## References

[1] Gaudet A.D., Popovich P.G., Ramer M.S. (2011). Wallerian degeneration: Gaining perspective on inflammatory events after peripheral nerve injury. J. Neuroinflammation.

[2] Ichihara S., Inada Y., Nakamura T. (2008). Artificial nerve tubes and their application for repair of peripheral nerve injury: An update of current concepts. Injury.

[3] Liao I.C., Wan H., Qi S., Cui C., Patel P., Sun W., Xu H. (2013). Preclinical evaluations of acellular biological conduits for peripheral nerve regeneration. J. Tissue Eng..

[4] Aikeremujiang M., Ao Q. (2015). Past, Present, and Future of Nerve Conduits in the Treatment of Peripheral Nerve Injury. Biomed. Res. Int.

[5] Paprottka F.J., Wolf P., Harder Y., Kern Y., Paprottka P.M., Machens H.G., Lohmeyer J.A. (2013). Sensory Recovery Outcome after Digital Nerve Repair in Relation to Different Reconstructive Techniques: Meta-Analysis and Systematic Review. Plast. Surg. Int..

[6] Sva B. (2020). Nerve guide conduits for peripheral nerve injury repair: A review on design, materials and fabrication methods—ScienceDirect. Acta Biomater..

[7] Sionkowska A. (2011). Current research on the blends of natural and synthetic polymers as new biomaterials: Review. Prog. Polym. Sci..

[8] Gan L., Zhao L., Zhao Y., Li K., Tong Z., Yi L., Wang X., Li Y., Tian W., He X. (2016). Cellulose/soy protein composite-based nerve guidance conduits with designed microstructure for peripheral nerve regeneration. J. Neural Eng..

[9] Gu X., Ding F., Yang Y., Jie L. (2011). Construction of tissue engineered nerve grafts and their application in peripheral nerve regeneration. Prog. Neurobiol..

[10] Si J., Yang Y., Xing X., Yang F., Shan P. (2019). Controlled degradable chitosan/collagen composite scaffolds for application in nerve tissue regeneration. Polym. Degrad. Stab..

[11] Lau Y.T., Kwok L.F., Tam K.W., Chan Y.S., Shum K.Y., Shea K.H. (2017). Genipin-treated chitosan nanofibers as a novel scaffold for nerve guidance channel design. Colloids Surf. B Biointerfaces.

[12] Li G., Xue C., Wang H., Yang X., Zhao Y., Zhang L., Yang Y. (2018). Spatially featured porous chitosan conduits with micropatterned inner wall and seamless sidewall for bridging peripheral nerve regeneration. Carbohydr. Polym..

[13] Jiang Z., Song Y., Qiao J., Yang Y., Zhang W., Liu W., Han B. (2019). Rat sciatic nerve regeneration across a 10-mm defect bridged by a chitin/CM-chitosan artificial nerve graft. Int. J. Biol. Macromol..

[14] Hu X., Huang J., Ye Z., Xia L., Li M., Lv B., Shen X., Luo Z. (2009). A novel scaffold with longitudinally oriented microchannels promotes peripheral nerve regeneration. Tissue Eng. Part A.

[15] Berns E.J., Sur S., Pan L., Goldberger J.E., Suresh S., Zhang S., Kessler J.A., Stupp S.I. (2014). Aligned neurite outgrowth and directed cell migration in self-assembled monodomain gels. Biomaterials.

[16] Shariatinia Z. (2018). Carboxymethyl chitosan: Properties and biomedical applications. Int. J. Biol. Macromol..

[17] He B., Wu F., Fan L., Li X.H., Liu Y., Liu Y.J., Ding W.J., Deng M., Zhou Y. (2018). Carboxymethylated chitosan protects Schwann cells against hydrogen peroxide-induced apoptosis by inhibiting oxidative stress and mitochondria dependent pathway. Eur. J. Pharmacol..

[18] Wang G., Lu G., Ao Q., Gong Y., Zhang X. (2010). Preparation of cross-linked carboxymethyl chitosan for repairing sciatic nerve injury in rats. Biotechnol. Lett..

[19] Lu G., Kong L., Sheng B., Wang G., Gong Y., Zhang X. (2007). Degradation of covalently cross-linked carboxymethyl chitosan and its potential application for peripheral nerve regeneration. Eur. Polym. J..

[20] Agarwal T., Narayan R., Maji S., Behera S., Kulanthaivel S., Maiti T.K., Banerjee I., Pal K., Giri S. (2016). Gelatin/Carboxymethyl chitosan based scaffolds for dermal tissue engineering applications. Int. J. Biol. Macromol..

[21] Zhu S., Ge J., Wang Y., Qi F., Ma T., Wang M., Yang Y., Liu Z., Huang J., Luo Z. (2014). A synthetic oxygen carrier-olfactory ensheathing cell composition system for the promotion of sciatic nerve regeneration. Biomaterials.

[22] Jin J., Min S.L., Ju H.L., Si H.P., Yang H.S. (2020). Micro-grooved nerve guidance conduits combined with microfiber for rat sciatic nerve regeneration. J. Ind. Eng. Chem..

[23] Sarker M.D., Naghieh S., Mcinnes A.D., Schreyer D.J., Chen X. (2018). Regeneration of Peripheral Nerves by Nerve Guidance Conduits: Influence of Design, Biopolymers, Cells, Growth Factors, and Physical Stimuli. Prog. Neurobiol..

[24] Xie W.M., Xu P.X., Wang W., Liu Q. (2002). Preparation and antibacterial activity of a water-soluble chitosan derivative. Carbohyd. Polym..

[25] Chang G., Dang Q., Liu C., Wang X., Song H., Gao H., Sun H., Zhang B., Cha D. (2022). Carboxymethyl chitosan and carboxymethyl cellulose based self-healing hydrogel for accelerating diabetic wound healing. Carbohydr. Polym..

[26] Neubrech F., Sauerbier M., Moll W., Seegmüller J., Heider S., Harhaus L., Bickert B., Kneser U., Kremer T. (2018). Enhancing the Outcome of Traumatic Sensory Nerve Lesions of the Hand by Additional Use of a Chitosan Nerve Tube in Primary Nerve Repair: A Randomized Controlled Bicentric Trial. Plast. Reconstr. Surg..

[27] Boecker A., Daeschler S.C., Kneser U., Harhaus L. (2019). Relevance and Recent Developments of Chitosan in Peripheral Nerve Surgery. Front. Cell Neurosci..

[28] Marcol W., Larysz-Brysz M., Kucharska M., Niekraszewicz A., Slusarczyk W., Kotulska K., Wlaszczuk P., Wlaszczuk A., Jedrzejowska-Szypulka H., Lewin-Kowalik J. (2011). Reduction of Post-Traumatic Neuroma and Epineural Scar Formation in Rat Sciatic Nerve by Application of Microcrystallic Chitosan. Microsurgery.

[29] Jessen K.R., Mirsky R. (2016). The repair Schwann cell and its function in regenerating nerves. J. Physiol..

[30] Park C.J., Park S.A., Yoon T.G., Lee S.J., Yum K.W., Kim H.J. (2005). Bupivacaine Induces Apoptosis via ROS in the Schwann Cell Line. J. Dent. Res..

[31] Zhao Z., Li X., Li Q. (2017). Curcumin accelerates the repair of sciatic nerve injury in rats through reducing Schwann cells apoptosis and promoting myelinization. Biomed. Pharmacother..

[32] Purves T.D., Middlemas A., Agthong S., Jude E.B., Tomlinson D.R. (2001). A role for mitogen-activated protein kinases in the etiology of diabetic neuropathy. Faseb. J. Off. Publ. Fed. Am. Soc. Exp. Biol..

[33] Luo X., Tao L., Peng L., Mo X., Chen H. (2012). Extracellular heat shock protein 72 protects schwann cells from hydrogen peroxide-induced apoptosis. J. Neurosci. Res..

[34] He B., Tao H.Y., Liu S.Q. (2014). Neuroprotective effects of carboxymethylated chitosan on hydrogen peroxide induced apoptosis in Schwann cells. Eur. J. Pharmacol..

[35] Chang W., Shah M.B., Lee P., Yu X. (2018). Tissue-engineered spiral nerve guidance conduit for peripheral nerve regeneration. Acta Biomater..

[36] Jiang M., Zhuge X., Yang Y., Gu X., Ding F. (2009). The promotion of peripheral nerve regeneration by chitooligosaccharides in the rat nerve crush injury model. Neurosci. Lett..

[37] Wang X., Wen H., Yong C., Jian Y., Jian W., Gu X. (2005). Dog sciatic nerve regeneration across a 30-mm defect bridged by a chitosan/PGA artificial nerve graft. Brain.

[38] Chamberlain L.J., Yannas I.V., Hsu H.P., Strichartz G., Spector M. (1998). Collagen-GAG substrate enhances the quality of nerve regeneration through collagen tubes up to level of autograft. Exp. Neurol..

[39] Marina M.B., Marie J.P., Birchall M.A. (2011). Laryngeal reinnervation for bilateral vocal fold paralysis. Curr. Opin. Otolaryngol..

[40] Yang Y., Chen X., Ding F., Zhang P., Liu J., Gu X. (2007). Biocompatibility evaluation of silk fibroin with peripheral nerve tissues and cells in vitro. Biomaterials.

[41] Jiang Z., Chi J., Li H., Wang Y., Liu W., Han B. (2021). Effect of chitosan oligosaccharide-conjugated selenium on improving immune function and blocking gastric cancer growth. Eur. J. Pharm..

[42] Jahromi H.K., Farzin A., Hasanzadeh E., Barough S.E., Mahmoodi N., Najafabadi M.R.H., Farahani M.S., Mansoori K., Shirian S., Ai J. (2020). Enhanced sciatic nerve regeneration by poly-L-lactic acid/multi-wall carbon nanotube neural guidance conduit containing Schwann cells and curcumin encapsulated chitosan nanoparticles in rat. Mater. Sci. Eng. C.

[43] Dong C., Qiao F., Hou W., Yang L., Lv Y. (2020). Graphene-based conductive fibrous scaffold boosts sciatic nerve regeneration and functional recovery upon electrical stimulation. Appl. Mater. Today.

