# Correction: Zhang et al. Nerve Regeneration Effect of a Composite Bioactive Carboxymethyl Chitosan-Based Nerve Conduit with a Radial Texture. *Molecules* 2022, *27*, 9039

**DOI:** 10.3390/molecules30183659

**Published:** 2025-09-09

**Authors:** Yijie Zhang, Zhiwen Jiang, Yanting Wang, Lixin Xia, Shuqin Yu, Hongjian Li, Wei Zhang, Wanshun Liu, Kai Shao, Baoqin Han

**Affiliations:** 1 Laboratory of Biochemistry and Biomedical Materials, College of Marine Life Sciences, Ocean University of China, Qingdao 266003, China; 15020050785@163.com (Y.Z.); jiangzhiwen@ouc.edu.cn (Z.J.); 17860751735@163.com (Y.W.); xialixinzzz@163.com (L.X.); yu.shuqin@outlook.com (S.Y.); 21180631156@stu.ouc.edu.cn (H.L.); zhangwei0515@outlook.com (W.Z.); wanshunliu@hotmail.com (W.L.); 2 Laboratory for Marine Drugs and Bioproducts, Pilot National Laboratory for Marine Science and Technology, Qingdao 266235, China; 3 Department of Central Laboratory, Qilu Hospital (Qingdao), Cheeloo College of Medicine, Shandong University, Qingdao 266035, China

In the original publication [1], there was a mistake in Figure 7 and graphical abstract. The authors regret that the transmission electron microscope image of the autograft group for 12 w (Figure 7a and graphical abstract) was an incorrect image (due to inadvertent confusion during manuscript preparation).

The authors state that the scientific conclusions are unaffected. This correction was approved by the Academic Editor. The original publication has also been updated. 

The corrected Figure 7 is as follows:

**Figure 7:**
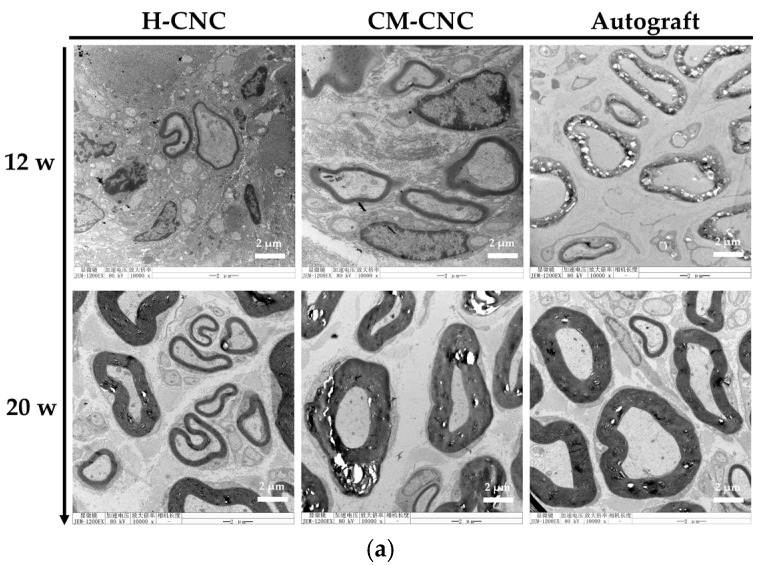

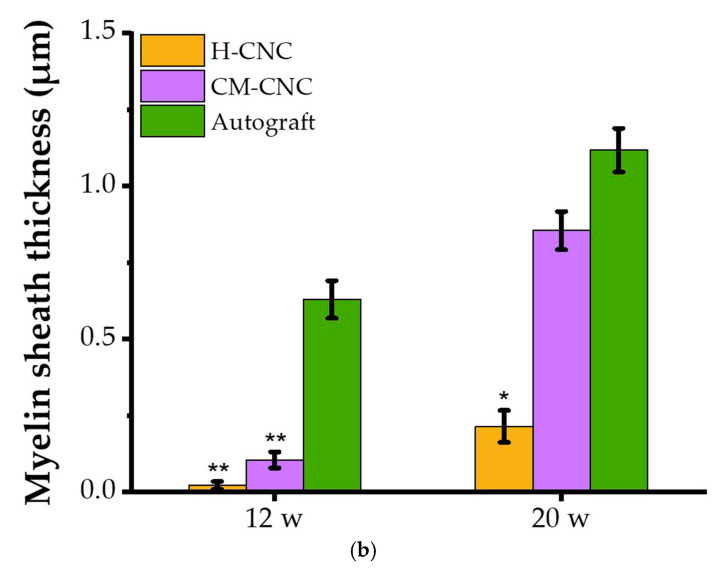
Effects of the CM-CNC on ultrastructural changes in regenerated nerves. (**a**) Observation of the ultrastructure of regenerated nerves through transmission electron microscopy (10,000×). (**b**) The thickness of the regenerated myelin sheath (μm). The data are represented by the mean ± SD, *n* = 6, * *p* < 0.05, ** *p* < 0.01 significant difference in comparison with the autograft group.

The corrected graphical abstract is as follows:


